# Comparation between novel online models and the AJCC 8th TNM staging system in predicting cancer-specific and overall survival of small cell lung cancer

**DOI:** 10.3389/fendo.2023.1132915

**Published:** 2023-07-25

**Authors:** Meiyun Liu, Peng Zhang, Suyu Wang, Wei Guo, Yibin Guo

**Affiliations:** ^1^ Department of Anesthesiology, Shanghai Pulmonary Hospital, School of Medicine, Tongji University, Shanghai, China; ^2^ Department of Cardiothoracic Surgery, The 961st Hospital of Joint Logistics Support Force of PLA, Qiqihar, China; ^3^ Department of Thoracic Surgery, Shanghai Pulmonary Hospital, School of Medicine, Tongji University, Shanghai, China; ^4^ Department of Health Statistics, Naval Medical University, Shanghai, China

**Keywords:** nomogram, small cell lung cancer, lymph node ratio, seer, survival

## Abstract

**Background:**

Most of previous studies on predictive models for patients with small cell lung cancer (SCLC) were single institutional studies or showed relatively low Harrell concordance index (C-index) values. To build an optimal nomogram, we collected clinicopathological characteristics of SCLC patients from Surveillance, Epidemiology, and End Results (SEER) database.

**Methods:**

24,055 samples with SCLC from 2010 to 2016 in the SEER database were analyzed. The samples were grouped into derivation cohort (n=20,075) and external validation cohort (n=3,980) based on America’s different geographic regions. Cox regression analyses were used to construct nomograms predicting cancer-specific survival (CSS) and overall survival (OS) using derivation cohort. The nomograms were internally validated by bootstrapping technique and externally validated by calibration plots. C-index was computed to compare the accuracy and discrimination power of our nomograms with the 8th of version AJCC TNM staging system and nomograms built in previous studies. Decision curve analysis (DCA) was applied to explore whether the nomograms had better clinical efficiency than the 8th version of AJCC TNM staging system.

**Results:**

Age, sex, race, marital status, primary site, differentiation, T classification, N classification, M classification, surgical type, lymph node ratio, radiotherapy, and chemotherapy were chosen as predictors of CSS and OS for SCLC by stepwise multivariable regression and were put into the nomograms. Internal and external validations confirmed the nomograms were accurate in prediction. C-indexes of the nomograms were relatively satisfactory in derivation cohort (CSS: 0.761, OS: 0.761) and external validation cohort (CSS: 0.764, OS: 0.764). The accuracy of the nomograms was superior to that of nomograms built in previous studies. DCA showed the nomograms conferred better clinical efficiency than 8th version of TNM staging system.

**Conclusions:**

We developed practical nomograms for CSS (https://guowei2020.shinyapps.io/DynNom-CSS-SCLC/) and OS (https://drboidedwater.shinyapps.io/DynNom-OS-SCLC/) prediction of SCLC patients which may facilitate clinicians in individualized therapeutics.

## Introduction

Lung cancer is a chief cause of death due to malignancy globally. Small cell lung cancer (SCLC), originated from neuroendocrine cells, is an aggressive cancer which accounts for approximately 15% of all lung cancers, causing 30,000 deaths annually (1). Unlike non-small cell lung cancer which showed excellent response to target therapy or immunotherapy (2–4), the recent clinical trials showed new drugs could only brought limited benefit for SCLC (5, 6). SCLC is characterized with high malignant level, high doubling rate, and early and extensive metastasis (7). The 5-year survival probability for patients with SCLC receiving no active treatment is as poor as less than 5% with an average overall survival (OS) time of merely 2-4 months (8, 9). National Comprehensive Cancer Network Clinical Practice Guidelines in Oncology (version 2.2018) suggest stage I SCLC patients receive surgery and adjuvant chemotherapy (10). However, more than 80% SCLC patients are identified at stage III/IV, causing a high mortality of SCLC (11). Most early-stage SCLC patients can benefit from platinum-based chemotherapy and radiation, while advanced or metastatic stage patients receive platinum-based chemotherapy alone (12). However, nearly all SCLC will recur ultimately because of early dissemination and acquired drug resistance.

Because of the heterogenous nature of SCLC, it needs to be dealt with as an individual entity. Considering that, the latest 8th version of American Joint Committee on Cancer (AJCC) tumor, lymph node, metastasis (TNM) staging system can predict the prognosis of SCLC more precisely than the 7th version (13). However, unlike the TNM staging systems for gastric and rectal cancer which take both anatomical regions and positive lymph nodes count into consideration, the TNM staging system for SCLC only include the anatomical regions of lymphatic metastasis (14, 15). Lymph node ratio (LNR) which is calculated by *LNR = number of positive lymph nodes/number of examined lymph nodes*, taking number of positive and examined lymph nodes into account, can solve this problem (16). What’s more, the TNM staging system doesn’t include the demographic data (age, sex, race, and marital status), histopathologic features (laterality, site, differentiation, and histology), and treatment modalities (surgery, radiotherapy, and chemotherapy) which may also be predictors for SCLC patients. Then it’s clear that the TNM staging system isn’t sufficient enough for the long-term survival prediction for SCLC patients. Therefore, an optimal model with better predictive performance is needed, and a nomogram is a satisfying tool to settle all these problems.

Nomogram is a visualized model to predict survival probability using multivariable Cox or other regression analyses of potential prediction variables. Most of previous studies on nomograms for SCLC patients were single center studies or showed relatively low Harrell concordance index (C-index) values which were the indicator for discrimination power and accuracy of prediction (17–20). In order to construct and validate a superior prognostic nomogram to help clinicians to choose treatment strategies, we performed this study. TRIPOD reporting checklist was used to guide the reporting of this research.

## Methods

### Data origin

The Surveillance, Epidemiology, and End Results (SEER) database contains patient information of 18 malignancy registries of the National Cancer Institute. As a national data bank, SEER covers information of approximately 30% of the US population (21). Clinicopathological information of SCLC patients was extracted from the SEER database (version 8.3.9; https://seer.cancer.gov/resources/). The 3rd edition of the International Classification of Diseases for Oncology was used to determine the primary site and histological type of the malignancy. The requirement for informed consent by patients and ethical approval by institutional review board were waived as all patient information in SEER database was deidentified before publication and included no information which could identify the patients. We performed this study in line with the Harmonized Tripartite Guideline for Good Clinical Practice from the International Conference on Harmonization and the Declaration of Helsinki (as revised in 2013).

### Patient screening

Altogether, 24,055 samples from SEER database were singled out for further analysis. Patients meeting following conditions were included: (I) primary lung cancer patients from 2010 to 2016 with primary site coding of C34.0-C34.9; (II) diagnosed with the histological type of small cell (ICD-O-3 codes: 8002, 8041-8045) with pathologic verification; (III) recognized as only one primary tumor. Samples with following criteria were excluded (I) were younger than 18 years; (II) had only autopsy or death certificate for diagnosis; (III) had missing information about race, marital status at diagnosis as well as laterality; (IV) had missing information concerning TNM staging system, examined lymph nodes number, positive lymph nodes number, surgical type, radiotherapy, chemotherapy as well as overall survival. The cancer stage of the study cohort was updated on the basis of the AJCC 8th TNM staging system (22).

Purchased/referred care delivery areas (PRCDA) was used to identify the geographic position of patients. Patients from East and Pacific Coast region of America were put into derivation cohort, while patients from PRCDA of Alaska, Northern Plains, and Southwest region were defined as external validation cohort. The nomogram was internally validated with bootstrapping technique in derivation cohort and externally validated by calibration plots in external validation cohort.

### Research variables and outcomes

Demographic data of the samples concerning age at diagnosis, sex, race, PRCDA region, and marital status at diagnosis was obtained. What’s more, histopathologic characteristics of cancer involving primary site, laterality, differentiation, histological type, T classification, N classification, M classification and LNR were extracted. The therapeutic regimens concerning surgical type, radiotherapy, and chemotherapy were also obtained. Continuous variables like age and LNR were changed into categorical variables. Age was categorized into <50, 50-59, 60-69, 70-79, and ≥80. Besides, for patients who underwent lymph node examination, LNR was dichotomized *via* the X-tile software (version 3.6.1; https://medicine.yale.edu/lab/rimm/research/software/) according to the cutoff value which could present the largest OS difference between two groups (23, 24).

In this study, we chose cancer-specific survival (CSS) and OS as the endpoints. CSS means the interval between diagnosis and SCLC-specific death, and OS means the interval between diagnosis and all-cause death with the unit of month. Follow-up data and survival outcome information from the SEER database updates every year and the latest ending date of follow-up information was December 31, 2016.

### Construction and evaluation of predictive model

To make the Cox model more accurate, continuous variables were changed into categorical variables and presented as count (percentage). Baseline features of samples in derivation and external validation cohort were put into comparation using standardized difference. Survival outcomes were presented with the Kaplan-Meier curves, and a two-sided log-rank test was used to detect the survival difference.

Moreover, univariable Cox analysis was applied to screen variables which could potentially predict the CSS or OS. Variables with P value for hazard ratio (HR) <0.1 selected by univariable Cox analysis were analyzed with multivariable Cox regression using stepwise Akaike Information Criterion (stepAIC) method to select the optimal predictors for the final models (25). Then, the HR with 95% confidence interval (CI) was reported. The ability of prediction of the model was evaluated according to discrimination, accuracy, and clinical efficiency. C-indexes were calculated to test the discrimination power of prediction, calibration and receiver operating characteristic (ROC) curves were plotted to test the accuracy, and decision curve analysis (DCA) was performed for assessment of the clinical efficiency (26–28).

### Establishment and validation of the nomogram

Variables screened out by multivariable Cox regression analysis using StepAIC method were put into the nomogram for CSS or OS of SCLC patients. Bootstrap technique was used for internal validation of the model with 1000 resamples of the derivation cohort. Calibration plots of 1-, 3-, and 5-year OS were utilized to compare the nomogram-predicted OS rate with the actual OS rate. The accuracy was considered to be high when the predictions fell closely to the diagonal line of the calibration plot.

We performed the statistical analyses by R software (version 4.1.0; http://www.r-project.org) and. The statistical tests were two-sided and statistical significance was achieved with P value smaller than 0.05.

## Results

### Baseline characteristics

From January 2010 to December 2016, 42,031 SCLC patients were reported in SEER database. After applying the screening criteria, 24,055 samples were retained as the study cohort. All of these had information relating to OS, and 23,883 of these had information relating to CSS. 20,075 patients (16,655 died before the last follow-up) from East and Pacific Coast region were put into the derivation cohort, and 3,980 patients (3,299 died before the last follow-up) from Alaska, Northern Plains, and Southwest region were defined as the external validation cohort. Process of sample screening was demonstrated in **Table 1**. Baseline characteristics of all samples grouped by derivation and external validation cohort were presented in **Table 2**. The whole study cohort’s median (interquartile range [IQR]) age at diagnosis was 67 years (60-74 years). The majority of the samples were diagnosed at the age of ≥50 years (96.2%), were white people (86.3%), were diagnosed at T3-4 classification (65.9%), N2-3 classification (77.8%), M1 classification (67.8%) and received chemotherapy (72.1%). Few patients (2.5%) received surgery, and nearly half patients (49.5%) received radiotherapy.

**Table 1:** Selection procedure of study cohort.

Step	Criteria	Number excluded	Number remained
1	Patients with SCLC between 2010 and 2016	–	42,031
2	Include if patients with only one primary tumor	9,611	32,420
3	Include if aged 18 or larger than 18 years	0	32,420
4	Exclude if patients only had diagnosis based on autopsy/death certificate	105	32,315
5	Missing information about race, marital status at diagnosis as well as laterality	3,544	28,771
6	Missing information about TNM staging, number of examined lymph nodes, number of positive lymph nodes, type of surgery, radiotherapy, chemotherapy as well as survival outcomes	4,716	24,055

SCLC, small cell lung cancer; TNM, tumor node metastasis.

**Table 2:** Baseline characteristics of derivation cohort and external validation cohort.

Characteristic	Total (n=24,055)		Derivation cohort (n=20,075)		External validation cohort (n=3,980)		Standardized difference
	N	%	N	%	N	%	
Age							0.064
<50	903	3.8	758	3.8	145	3.6	
50-59	4,866	20.2	3,989	19.9	877	22	
60-69	8,720	36.3	7,340	36.6	1,380	34.7	
70-79	6,970	29	5,847	29.1	1,123	28.2	
≥80	2,596	10.8	2,141	10.7	455	11.4	
Sex							0.007
Male	12,043	50.1	10,039	50	2,004	50.4	
Female	12,012	49.9	10,036	50	1,976	49.6	
Race							0.145
White	20,771	86.3	17,215	85.8	3,556	89.3	
Black	2,243	9.3	1,908	9.5	335	8.4	
Other	1,041	4.3	952	4.7	89	2.2	
Marital status							0.113
Married	11,987	49.8	9,967	49.6	2,020	50.8	
Separated	354	1.5	323	1.6	31	0.8	
Unmarried	3,790	15.8	3,243	16.2	547	13.7	
Divorced	3,756	15.6	3,063	15.3	693	17.4	
Widowed	4,168	17.3	3,479	17.3	689	17.3	
Laterality							0.015
Right	13,876	57.7	11,556	57.6	2,320	58.3	
Left	10,179	42.3	8,519	42.4	1,660	41.7	
Site							0.088
Main bronchus	2,854	11.9	2,455	12.2	399	10	
Upper lobe	12,289	51.1	10,189	50.8	2,100	52.8	
Middle lobe	980	4.1	825	4.1	155	3.9	
Lower lobe	5,128	21.3	4,248	21.2	880	22.1	
Over lapping lesion of lung	366	1.5	291	1.4	75	1.9	
Unknown	2,438	10.1	2,067	10.3	371	9.3	
Differentiation							0.14
Grade I	33	0.1	27	0.1	6	0.2	
Grade II	66	0.3	61	0.3	5	0.1	
Grade III	2,141	8.9	1,885	9.4	256	6.4	
Grade IV	3,336	13.9	2,855	14.2	481	12.1	
Unknown	18,479	76.8	15,247	76	3,232	81.2	
Histology							0.116
Oat cell	275	1.1	260	1.3	15	0.4	
Fusiform cell	16	0.1	16	0.1	0	0	
Intermediate cell	43	0.2	40	0.2	3	0.1	
Combined small cell	439	1.8	355	1.8	84	2.1	
Unknown	23,282	96.8	19,404	96.7	3,878	97.4	
T classification							0.064
T1	3,117	13	2,643	13.2	474	11.9	
T2	5,097	21.2	4,173	20.8	924	23.2	
T3	4,706	19.6	3,940	19.6	766	19.2	
T4	11,135	46.3	9,319	46.4	1,816	45.6	
N classification							0.085
N0	3,550	14.8	3,006	15	544	13.7	
N1	1,791	7.4	1,472	7.3	319	8	
N2	13,373	55.6	11,244	56	2,129	53.5	
N3	5,341	22.2	4,353	21.7	988	24.8	
M classification							0.086
M0	7,738	32.2	6,589	32.8	1,149	28.9	
M1	16,317	67.8	13,486	67.2	2,831	71.1	
Surgery							0.045
Not performed	23,458	97.5	19,567	97.5	3,891	97.8	
Sublobectomy	237	1	199	1	38	1	
Lobectomy	342	1.4	291	1.4	51	1.3	
Pneumonectomy	18	0.1	18	0.1	0	0	
LN examination							0.19
No LN examined	18,304	76.1	15,471	77.1	2,833	71.2	
0≤LNR<0.6	827	3.4	701	3.5	126	3.2	
0.6≤LNR ≤ 1	786	3.3	691	3.4	95	2.4	
LNR>0, NOS	4,138	17.2	3,212	16	926	23.3	
Radiotherapy							0.052
No	12,155	50.5	10,230	51	1,925	48.4	
Yes	11,900	49.5	9,845	49	2,055	51.6	
Chemotherapy							0.072
No	6,710	27.9	5,705	28.4	1,005	25.3	
Yes	17,345	72.1	14,370	71.6	2,975	74.7	

Standardized difference was calculated by comparing the variables of derivation cohort and external validation cohort. LN, lymph node; LNR, lymph node ratio; NOS, not otherwise specified.

### Variable selection and survival analysis

**Figure 1:**
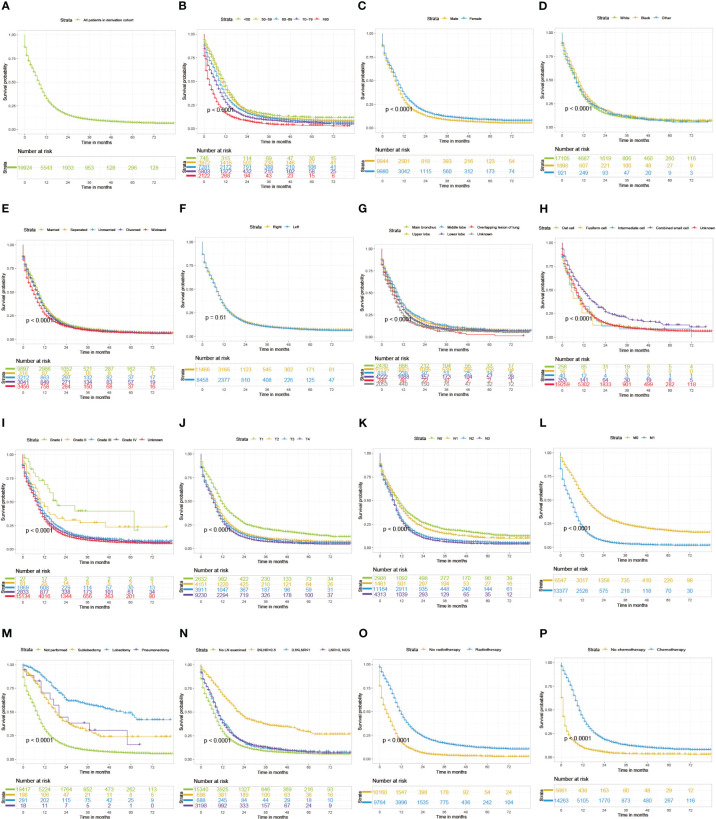
Kaplan-Meier curves of CSS for SCLC patients grouped by all patients **(A)**, age **(B)**, sex **(C)**, race **(D)**, marital status **(E)**, laterality **(F)**, site **(G)**, differentiation **(H)**, Histology **(I)**, T classification **(J)**, N classification **(K)**, M classification **(L)**, surgery **(M)**, lymph node ratio **(N)**, radiotherapy **(O)**, and chemotherapy **(P)**.CSS, cancer-specific survival; SCLC, small cell lung cancer; LN, lymph node; LNR, lymph node ratio.

**Figure 2:**
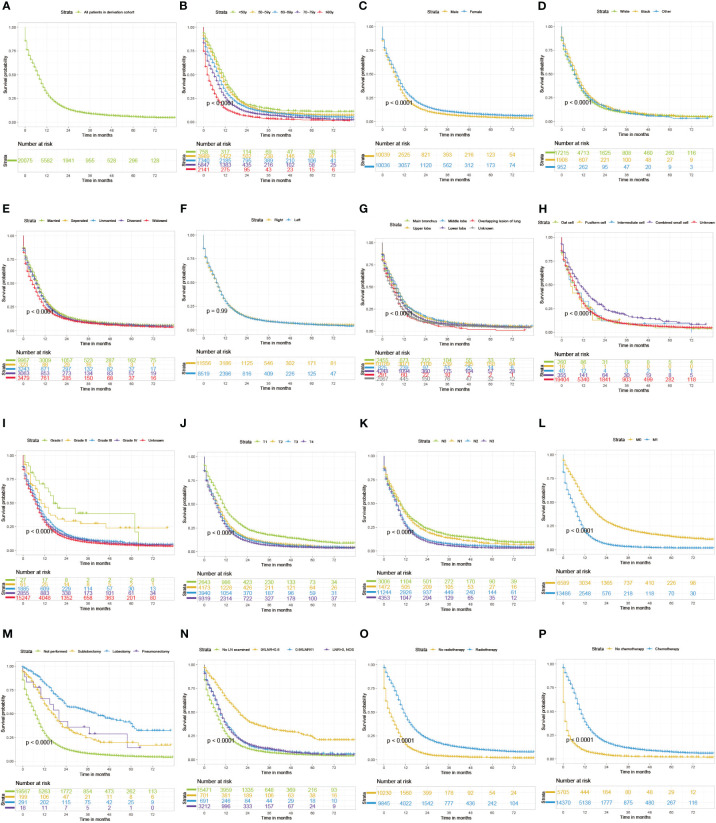
Kaplan-Meier curves of OS for SCLC patients grouped by all patients **(A)**, age **(B)**, sex **(C)**, race **(D)**, marital status **(E)**, laterality **(F)**, site **(G)**, differentiation **(H)**, Histology **(I)**, T classification **(J)**, N classification **(K)**, M classification **(L)**, surgery **(M)**, lymph node ratio **(N)**, radiotherapy **(O)**, and chemotherapy **(P)**. OS, overall survival; SCLC, small cell lung cancer; LN, lymph node; LNR, lymph node ratio.

LNR was classified into 0≤LNR<0.6 and 0.6≤LNR ≤ 1 using X-tile software (**Supplementary Figure 1**). The median (IQR) follow-up months of derivation cohort was 41 months (20-62 months). The cumulative 1-, 3-, and 5-year CSS of the derivation cohort were 32.4% (95% CI, 31.7-33.1%), 10.2% (95% CI, 9.7-10.7%), and 7.2% (95% CI, 6.7-7.7%) respectively. And the cumulative 1-, 3-, and 5-year OS of the derivation cohort were 30.1% (95% CI, 29.5-30.8%), 8.8% (95% CI, 8.4-9.3%), and 5.6% (95% CI, 5.2-6.1%) respectively. As displayed in **Figures 1**, **2**, patients with older age, male sex, white race, unmarried/divorced/widowed status, unknown primary site, overlapping lesion, higher grade of differentiation, histological type of non-combined small cell, more progressive T classification, more progressive N classification, more progressive M classification, higher LNR value, no surgery, no radiotherapy, and no chemotherapy had significantly worse CSS or OS probabilities (log-rank test P value <0.01). Univariable Cox analysis revealed that age at diagnosis, sex, race, marital status, primary site, differentiation, histological type, T classification, N classification, M classification, surgical type, lymph node ratio, radiotherapy, and chemotherapy were significantly correlated with OS (P value <0.1) in **Tables 3**, **4**. Thereafter, above variables were put into multivariable Cox analysis with the method of StepAIC. The results exhibited that age at diagnosis, sex, race, marital status, primary site, differentiation, T classification, N classification, M classification, surgical type, lymph node ratio, radiotherapy, and chemotherapy were chosen to construct the final models (**Tables 3**, **4**).

**Table 3:** Univariable and StepAIC multivariable Cox regression analysis for CSS.

Characteristic	Univariable analysis		Multivariable analysis	
	HR (95% CI)	P	HR (95% CI)	P
Age				
<50	1		1	
50-59	1.081 (0.989-1.183)	0.087	1.039 (0.949-1.136)	0.408
60-69	1.247 (1.144-1.359)	<0.001	1.152 (1.057-1.257)	0.001
70-79	1.528 (1.401-1.668)	<0.001	1.305 (1.194-1.426)	<0.001
≥80	2.275 (2.070-2.500)	<0.001	1.565 (1.418-1.727)	<0.001
Sex				
Male	1		1	
Female	0.855 (0.829-0.883)	<0.001	0.867 (0.839-0.896)	<0.001
Race				
White	1		1	
Black	0.873 (0.827-0.922)	<0.001	0.842 (0.796-0.890)	<0.001
Other	0.950 (0.879-1.026)	0.189	0.915 (0.847-0.988)	0.024
Marital status				
Married	1		1	
Separated	1.048 (0.922-1.191)	0.471	1.197 (1.052-1.361)	0.006
Unmarried	1.072 (1.024-1.122)	0.003	1.109 (1.058-1.162)	<0.001
Divorced	1.062 (1.014-1.112)	0.011	1.124 (1.072-1.177)	<0.001
Widowed	1.237 (1.184-1.292)	<0.001	1.116 (1.064-1.170)	<0.001
Laterality				
Right	1			
Left	1.008 (0.976-1.041)	0.621		
Site				
Main bronchus	1		1	
Upper lobe	0.873 (0.831-0.918)	<0.001	0.956 (0.909-1.006)	0.082
Middle lobe	0.862 (0.787-0.944)	0.001	0.941 (0.859-1.031)	0.191
Lower lobe	0.971 (0.919-1.028)	0.312	1.000 (0.944-1.058)	0.988
Over lapping lesion of lung	1.170 (1.022-1.338)	0.022	0.999 (0.874-1.143)	0.993
Unknown	1.162 (1.088-1.240)	<0.001	1.061 (0.993-1.133)	0.079
Differentiation				
Grade I	1		1	
Grade II	1.491 (0.824-2.700)	0.187	1.456 (0.803-2.638)	0.216
Grade III	2.113 (1.270-3.514)	0.004	2.003 (1.203-3.336)	0.008
Grade IV	2.309 (1.390-3.837)	0.001	2.058 (1.237-3.424)	0.005
Unknown	2.508 (1.512-4.162)	<0.001	2.084 (1.254-3.463)	0.005
Histology				
Oat cell	1			
Fusiform cell	1.087 (0.608-1.944)	0.778		
Intermediate cell	1.036 (0.722-1.488)	0.847		
Combined small cell	0.683 (0.569-0.820)	<0.001		
Unknown	0.991 (0.866-1.133)	0.89		
T classification				
T1	1		1	
T2	1.438 (1.356-1.525)	<0.001	1.261 (1.188-1.338)	<0.001
T3	1.530 (1.442-1.623)	<0.001	1.307 (1.230-1.388)	<0.001
T4	1.668 (1.583-1.758)	<0.001	1.307 (1.237-1.381)	<0.001
N classification				
N0	1		1	
N1	1.095 (1.017-1.179)	0.016	1.201 (1.115-1.294)	<0.001
N2	1.445 (1.377-1.516)	<0.001	1.476 (1.404-1.553)	<0.001
N3	1.502 (1.422-1.587)	<0.001	1.493 (1.408-1.584)	<0.001
M classification				
M0	1		1	
M1	2.568 (2.476-2.664)	<0.001	2.170 (2.087-2.256)	<0.001
Surgery				
Not performed	1		1	
Sublobectomy	0.439 (0.363-0.530)	<0.001	0.619 (0.509-0.753)	<0.001
Lobectomy	0.214 (0.176-0.260)	<0.001	0.383 (0.305-0.481)	<0.001
Pneumonectomy	0.438 (0.249-0.772)	0.004	0.649 (0.363-1.160)	0.145
LN examination				
No LN examined	1		1	
0≤LNR<0.6	0.360 (0.324-0.401)	<0.001	0.725 (0.639-0.823)	<0.001
0.6≤LNR ≤ 1	0.775 (0.711-0.846)	<0.001	0.905 (0.829-0.988)	0.025
LNR>0, NOS	0.768 (0.734-0.803)	<0.001	0.883 (0.843-0.925)	<0.001
Radiotherapy				
No	1		1	
Yes	0.425 (0.411-0.439)	<0.001	0.642 (0.619-0.664)	<0.001
Chemotherapy				
No	1		1	
Yes	0.292 (0.282-0.303)	<0.001	0.307 (0.295-0.319)	<0.001

stepAIC, stepwise Akaike information criterion; CSS, cancer-specific survival; HR, hazard ratio; CI, confidence interval; LN, lymph node; LNR, lymph node ratio; NOS, not otherwise specified.

**Table 4:** Univariable and StepAIC multivariable Cox regression analysis for OS.

Characteristic	Univariable analysis		Multivariable analysis	
	HR (95% CI)	P	HR (95% CI)	P
Age				
<50	1		1	
50-59	1.110 (1.017-1.212)	0.019	1.073 (0.983-1.172)	0.116
60-69	1.289 (1.185-1.403)	<0.001	1.201 (1.103-1.307)	<0.001
70-79	1.606 (1.475-1.749)	<0.001	1.376 (1.262-1.501)	<0.001
≥80	2.391 (2.180-2.621)	<0.001	1.638 (1.488-1.802)	<0.001
Sex				
Male	1		1	
Female	0.850 (0.824-0.876)	<0.001	0.856 (0.829-0.883)	<0.001
Race				
White	1		1	
Black	0.886 (0.841-0.934)	<0.001	0.855 (0.810-0.902)	<0.001
Other	0.962 (0.895-1.035)	0.297	0.916 (0.851-0.986)	0.019
Marital status				
Married	1		1	
Separated	1.073 (0.950-1.212)	0.258	1.233 (1.090-1.394)	<0.001
Unmarried	1.099 (1.052-1.148)	<0.001	1.141 (1.090-1.194)	<0.001
Divorced	1.071 (1.024-1.119)	0.003	1.136 (1.086-1.188)	<0.001
Widowed	1.264 (1.212-1.319)	<0.001	1.128 (1.078-1.181)	<0.001
Laterality				
Right	1			
Left	1.000 (0.969-1.031)	0.977		
Site				
Main bronchus	1		1	
Upper lobe	0.886 (0.844-0.930)	<0.001	0.964 (0.918-1.012)	0.141
Middle lobe	0.886 (0.812-0.967)	0.007	0.958 (0.877-1.046)	0.336
Lower lobe	0.985 (0.933-1.040)	0.597	1.008 (0.954-1.065)	0.775
Over lapping lesion of lung	1.192 (1.048-1.357)	0.008	1.030 (0.905-1.172)	0.658
Unknown	1.174 (1.102-1.251)	<0.001	1.076 (1.010-1.147)	0.023
Differentiation				
Grade I	1		1	
Grade II	1.307 (0.741-2.306)	0.355	1.247 (0.706-2.202)	0.446
Grade III	2.001 (1.241-3.228)	0.004	1.902 (1.178-3.072)	0.009
Grade IV	2.177 (1.351-3.507)	0.001	1.961 (1.215-3.164)	0.006
Unknown	2.360 (1.467-3.798)	<0.001	1.984 (1.231-3.198)	0.005
Histology				
Oat cell	1			
Fusiform cell	1.009 (0.565-1.802)	0.976		
Intermediate cell	0.958 (0.668-1.372)	0.813		
Combined small cell	0.690 (0.579-0.822)	<0.001		
Unknown	0.983 (0.865-1.118)	0.797		
T classification				
T1	1		1	
T2	1.394 (1.318-1.474)	<0.001	1.239 (1.170-1.311)	<0.001
T3	1.465 (1.384-1.550)	<0.001	1.271 (1.200-1.347)	<0.001
T4	1.581 (1.504-1.663)	<0.001	1.262 (1.198-1.330)	<0.001
N classification				
N0	1		1	
N1	1.059 (0.987-1.137)	0.113	1.161 (1.081-1.247)	<0.001
N2	1.386 (1.324-1.451)	<0.001	1.442 (1.374-1.513)	<0.001
N3	1.440 (1.367-1.517)	<0.001	1.469 (1.389-1.554)	<0.001
M classification				
M0	1		1	
M1	2.406 (2.324-2.491)	<0.001	2.042 (1.967-2.119)	<0.001
Surgery				
Not performed	1		1	
Sublobectomy	0.471 (0.395-0.563)	<0.001	0.636 (0.529-0.764)	<0.001
Lobectomy	0.246 (0.206-0.293)	<0.001	0.410 (0.333-0.506)	<0.001
Pneumonectomy	0.445 (0.258-0.767)	0.004	0.653 (0.374-1.141)	0.135
LN examination				
No LN examined	1		1	
0≤LNR<0.6	0.384 (0.347-0.424)	<0.001	0.732 (0.649-0.826)	<0.001
0.6≤LNR ≤ 1	0.772 (0.710-0.839)	<0.001	0.901 (0.828-0.981)	0.016
LNR>0, NOS	0.768 (0.736-0.802)	<0.001	0.883 (0.845-0.924)	<0.001
Radiotherapy				
No	1		1	
Yes	0.420 (0.407-0.433)	<0.001	0.629 (0.608-0.651)	<0.001
Chemotherapy				
No	1		1	
Yes	0.286 (0.277-0.296)	<0.001	0.307 (0.296-0.319)	<0.001

stepAIC, stepwise Akaike information criterion; OS, overall survival; HR, hazard ratio; CI, confidence interval; LN, lymph node; LNR, lymph node ratio; NOS, not otherwise specified.

### Establishment and validation of nomogram

**Figure 3:**
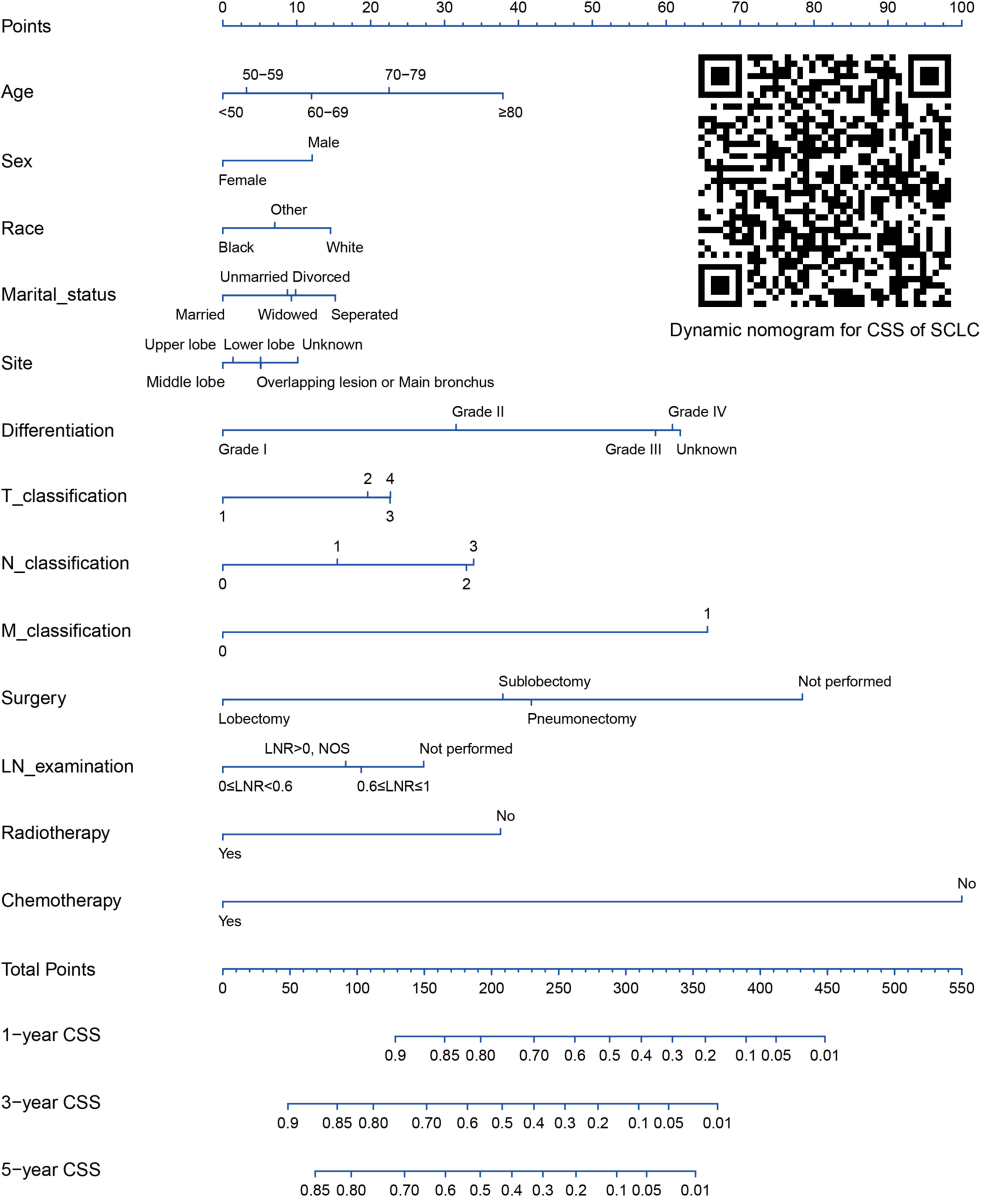
Nomogram for 1-, 3- and 5-year CSS prediction of SCLC patients. CSS, cancer-specific survival; SCLC, small cell lung cancer; LN, lymph node; LNR, lymph node ratio; NOS, not otherwise specified.

**Figure 4:**
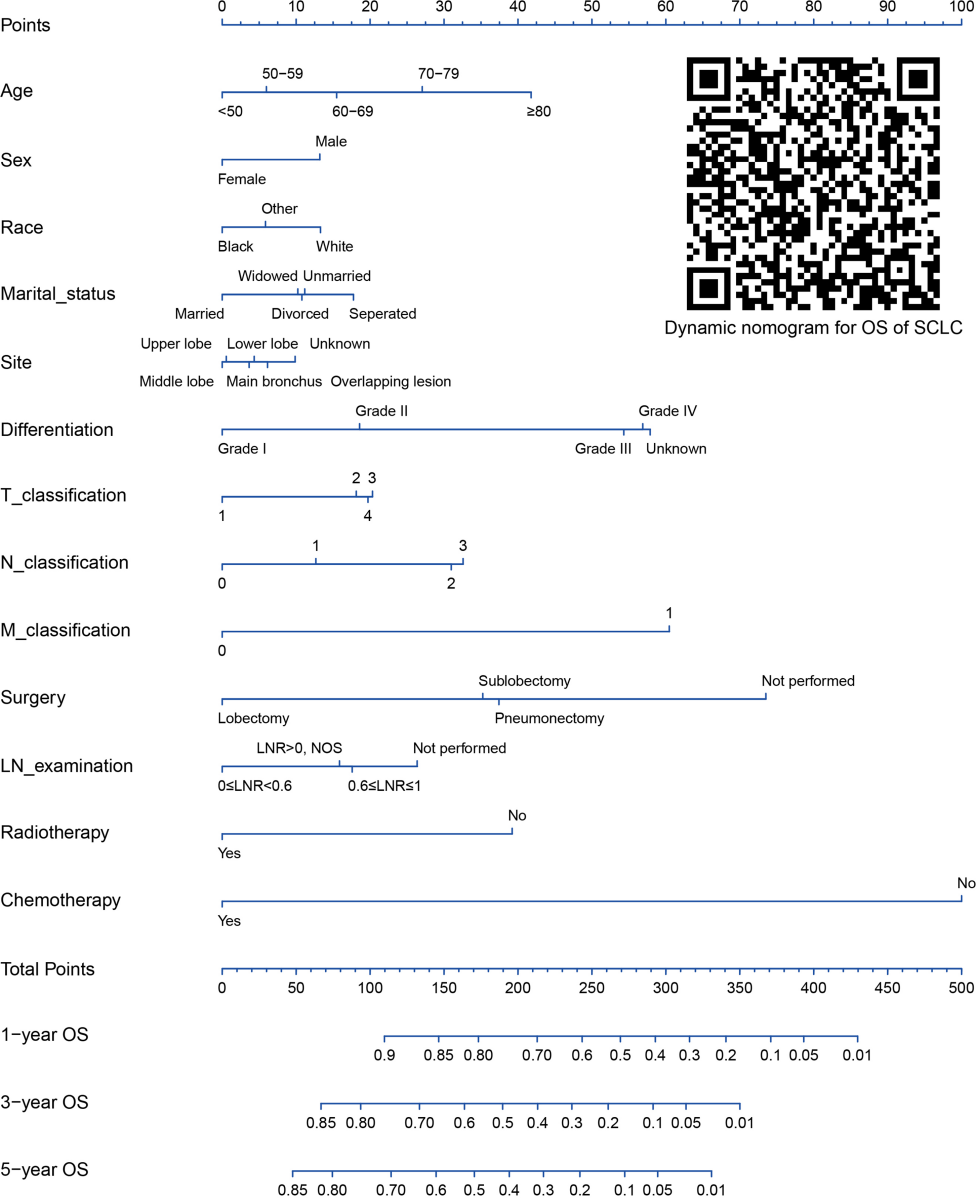
Nomogram for 1-, 3- and 5-year OS prediction of SCLC patients. OS, overall survival; SCLC, small cell lung cancer; LN, lymph node; LNR, lymph node ratio; NOS, not otherwise specified.

Variables screened by multivariable Cox analysis using StepAIC method were incorporated into nomograms (**Figures 3**, **4**). As shown in the nomograms, chemotherapy and surgery exert the greatest impact on the nomograms, followed by M classification, differentiation, age at diagnosis, and other variables. Each factor of the variables in the nomograms could be translated into to a score on the point scale. And the total points computed by adding the corresponding points of variables and the estimated CSS or OS could be acquired through plotting a straight line down from the total points.

**Figure 5:**
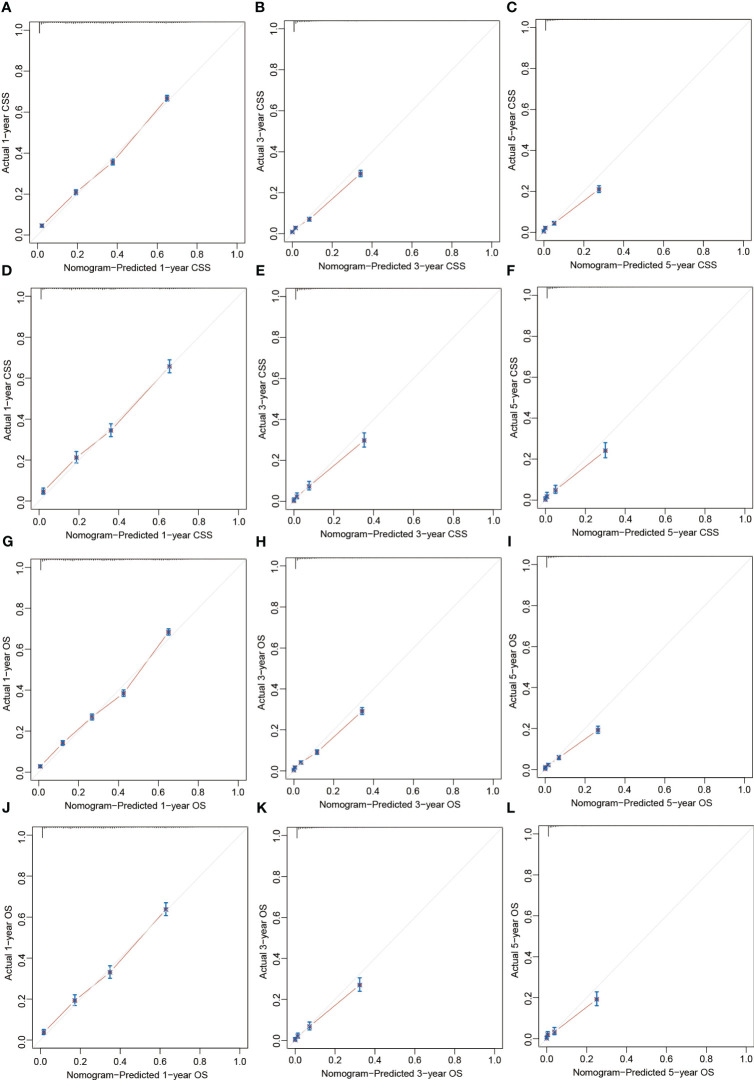
Calibration plots of the nomograms predicting 1-, 3-, and 5-year CSS in the derivation cohort **(A-C)** and validation cohort **(D-F)** and 1-, 3-, and 5-year OS in the derivation cohort **(G-I)** and external validation cohort **(J-L)**. CSS, cancer-specific survival; OS, overall survival.

The C-indexes of our nomograms were both 0.761 (95% CI: 0.757-0.765) for CSS and OS. The internal validation by bootstrapping technique with 1000 replicated sampling of the derivation cohort suggested the adjusted C-index for the nomograms were both 0.760, suggesting a sound predictive ability. As for the external validation, the C-indexes of the nomograms in the external validation cohort were both 0.764 (95% CI: 0.755-0.774) for CSS and OS which were even better than those of the derivation cohort. In addition, the calibration curves exhibited the predictions fell close to the diagonal line, demonstrating an ideal conformity between predicted and actual CSS or OS rates (**Figure 5**). Based on all of the above, the prediction of the nomogram is convincingly accurate.

### Comparation on predictive power of the 8th version of AJCC TNM and Nomogram

**Figure 6:**
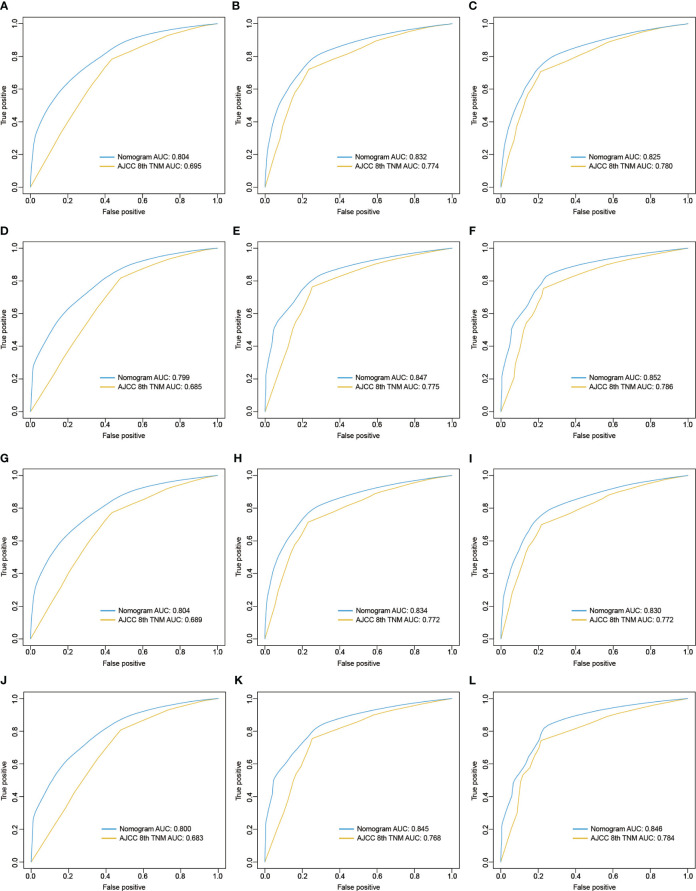
ROC curves of the 8th version of TNM staging system and nomogram for predicting 1-, 3-, and 5-year CSS in the derivation cohort **(A-C)** and validation cohort **(D-F)** and 1-, 3-, and 5-year OS in the derivation cohort **(G-I)** and validation cohort **(J-L)**. ROC, receiver operating characteristic; AJCC, American Joint Committee on Cancer; TNM, tumor, node, metastasis; CSS, cancer-specific survival; OS, overall survival.

**Figure 7:**
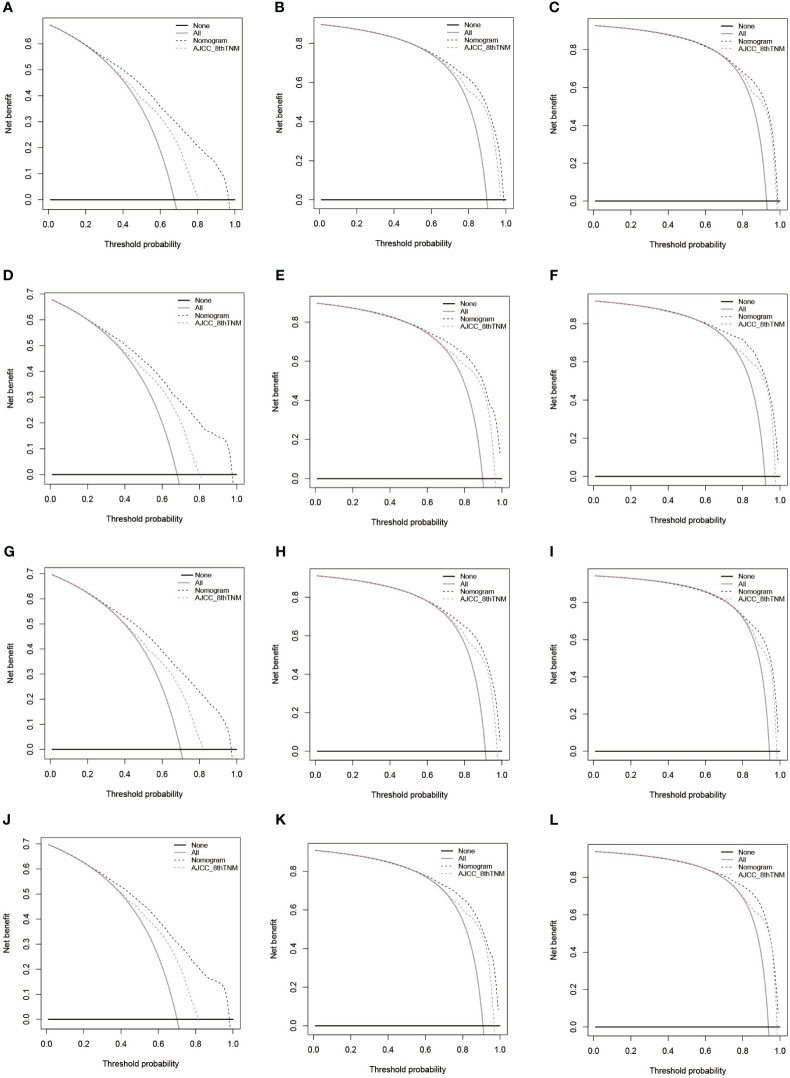
DCA of the 8th version of TNM staging system and nomogram for predicting 1-, 3-, and 5-year CSS in the derivation cohort **(A-C)** and validation cohort **(D-F)** and 1-, 3-, and 5-year OS in the derivation cohort **(G-I)** and validation cohort **(J-L)**. DCA, decision curve analysis; AJCC, American Joint Committee on Cancer; TNM, tumor, node, metastasis; CSS, cancer-specific survival; OS, overall survival.

The C-indexes of the 8th AJCC TNM staging system in derivation cohort [0.622 (95% CI: 0.616-0.627) for CSS and 0.614 (95% CI: 0.609-0.619) for OS] and external validation cohort [0.617 (95% CI: 0.605-0.628) for CSS and 0.613 (95% CI: 0.602-0.625) for OS] were inferior to those of the nomograms. In addition, the areas under ROC curve of the nomograms were higher than the 8th AJCC TNM staging system for predicting 1-, 3-, and 5-year CSS or OS in both derivation and external validation cohorts, indicating a better prediction accuracy (**Figure 6**). And DCA plots for CSS or OS demonstrated that the nomogram had a good performance of clinical efficiency, and patients might benefit more from the nomogram than the TNM staging system for both the derivation and validation cohorts (**Figure 7**).

### Construction of a webserver of the nomogram

Online dynamic nomograms based on our study were constructed for CSS (https://guowei2020.shinyapps.io/DynNom-CSS-SCLC/) and OS (https://drboidedwater.shinyapps.io/DynNom-OS-SCLC/) prediction. By typing in the variables, predicted CSS or OS rate with 95% CI and Kaplan-Meier curve can be output in the webserver. Two quick response (QR) codes were provided in **Figures 3**, **4** which could make the usage convenient.

## Discussion

In this multicenter study, SCLC patients’ data was extracted from the SEER database according to the screening criteria, and univariable and multivariable Cox regression analyses were employed to detect the independent predictors for SCLC. Two nomograms were built up using the selected variables and validated internally and externally. Comparing to the 8th TNM staging system, the nomograms demonstrated higher accuracy and superior clinical efficiency. Besides, we developed two webservers for clinicians to conveniently perform individual survival prediction. Visualization of Cox regression analyses proved these 13 variables are of significant influence on prognosis: age, sex, race, marital status, primary site, differentiation, T classification, N classification, M classification, surgical type, lymph node ratio, radiotherapy, and chemotherapy. Older age means more degenerative changes in organs function and prevalence of more comorbidities which cause worse outcome (29). Male patients showed worse survival than female ones, which could be seen in three other studies (16, 19, 20). White patients exhibited worse survival outcome compared with Black, which was similar with results of two previous studies (20, 30). As for marital status, our study revealed that married SCLC patients had better survival than other marital statuses, which wasn’t found in other studies on SCLC. While, studies on non-small cell lung cancer suggested marital status was correlated with survival outcome (27, 31). The survival benefit might be brought by financial and emotional support from the spouses.

80-90% SCLC patients are diagnosed at stage III-IV disease, and the Staging and Prognostic Factors Committee Advisory Boards and Participating Institutions have certified the predictive ability of clinical and pathological TNM staging for SCLC patients and suggested continuous application of the 8th version of AJCC TNM staging system for lung cancer in SCLC (32, 33). However, using only TNM staging system is not enough for individual prognosis prediction. The nodal staging of 8th AJCC TNM staging system rests on the concept that lymphatic metastasis starts in the nodes nearest to the primary malignancy and metastasize to nodes far from the tumor afterwards (34). N classification defines N1 as metastasis to ipsilateral peribronchial and/or hilar nodes and intrapulmonary nodes, N2 as metastasis to ipsilateral mediastinal and/or subcarinal nodes, and N3 as metastasis to contralateral mediastinum, contralateral hilar, ipsilateral or contralateral anterior scalene and supraclavicular nodes, without taking number of examined or positive lymph nodes into account (35). In order to solve this problem, we added the dichotomized LNR value into the models, and the optimal cut-off value was 0.6 calculated by the X-tile software, while in Wang’s study, the cut-off value was 0.01 (16). This may because Wang’s study cohort was with early stage and resected SCLC, and our study cohort included unresectable SCLC patients who underwent lymph node biopsy. In fact, our nomogram is fit for all T1-4N0-3M0-1 SCLC patients.

We searched previous studies on nomogram for SCLC on PubMed. Xie’s group introduced two nomograms predicting OS for SCLC patients incorporating pretreatment peripheral blood markers including ratios of inflammatory cell counts and red cell distribution width. Xie’s nomograms were based on a single institutional study with 938 patients and had lower C-index than ours (0.73 *vs*. 0.761), what’s more, Xie’s group built two nomograms for patients with extensive stage and limited stage SCLC while our one nomogram for OS can be used in all SCLC patients (18). Pan’s and Xiao’s nomograms for OS of SCLC were also developed from single center with lower C-indexes than ours (0.68 *vs*. 0.761, 0.60 *vs*. 0.761) (17, 19). The recent study of nomogram for SCLC conducted by Wang used clinical data of 24,680 patients from National Cancer Database (20). However, comparing to Wang’s nomogram, ours presented a better discrimination power (C-index: 0.761 *vs*. 0.722), maybe because of deficiency of information of site, differentiation, and detailed T, N, and M classification data of Wang’s nomogram.

As for the treatment modality, the surgery group exhibited survival benefit, and patients underwent lobectomy benefited from surgery most. However, only 2.6% of the overall study cohort received surgery. The poor effectiveness of therapeutic strategies for SCLC progression nowadays is connected with the lack of early diagnosis, and these patients usually have no chance of receiving surgery, because distant metastasis or paraneoplastic syndrome detracts the therapeutic potential of surgery (36). Patients with early-stage SCLC after surgery had improved survival was manifested by some previous studies (10, 12, 30). First-line standard chemotherapy for SCLC is combining etoposide or irinotecan with platinum. Concurrent or sequential radiotherapy is needed for limited stage disease, while chemotherapy serves as the mainstream strategy in the first-line setting (37). As we can see in **Figures 3**, **4**, chemotherapy made the largest contribution to our nomogram, indicating the great importance of chemotherapy for SCLC. Moreover, our study revealed most SCLC patients (72.1%) received chemotherapy and nearly half (49.5%) received radiotherapy, which is consistent with Wang’s large cohort study (20). Targeted therapy and immunotherapy have also been deeply researched and showed some encouraging results in recent years, however, the treatment modalities information screened from SEER database didn’t include targeted therapy and immunotherapy (5, 6, 37, 38).

As far as we know, our study, as a multicenter research with a large study cohort, introduces nomograms with the highest C-index indicating highest discrimination power and accuracy for prediction of prognosis for all-stage SCLC. Several limitations have to be admitted in this research. First, it’s a retrospective study, making it susceptible to the inherent weakness of retrospective data collection. Second, SEER database is short of some variables potentially influencing CSS or OS, such as smoking history, laboratory test results such as neutrophil or lymphocyte count, platelet count, red cell distribution width, and tumor markers associated with SCLC, etc. Third, detailed treatment modalities can’t be found in SEER database such as sequence between chemotherapy and surgery/radiotherapy, specific radiotherapy, chemotherapy, target therapy or immunotherapy regimens, thus making the risk scores of different therapeutic strategies can’t be presently applied as a guideline for regimen choice, because clinical therapeutic modalities need to be chosen according to all the covariables which influence the survival outcome.

## Conclusions

We constructed and validated nomograms for CSS and OS of SCLC, which demonstrated superior prediction performance to AJCC 8th TNM staging system or nomograms built in previous studies. Webservers was built based on the nomogram which may help clinicians in decision-making.

## Data availability statement

The raw data supporting the conclusions of this article will be made available by the authors, without undue reservation.

## Ethics statement

Ethical review and approval was not required for the study on human participants in accordance with the local legislation and institutional requirements. Written informed consent for participation was not required for this study in accordance with the national legislation and the institutional requirements.

## Author contributions

Conception/design: ML, PZ, and YG. Collection and/or assembly of data: ML, PZ, SW, and WG. Data analysis and interpretation: PZ and SW. Manuscript writing: SW, WG, and YG. Funding support: YG. All authors contributed to the article and approved the submitted version.

## Supplementary material

Supplementary Figure 1 The interface of X-tile categorizing LNR to achieve the optimal cut-off value. LNR, lymph node ratio.
