# Supplementary figures and images for: Comparation between novel online models and the AJCC 8th TNM staging system in predicting cancer-specific and overall survival of small cell lung cancer
Source: Front Endocrinol (Lausanne). 2023 Jul 25;14:1132915. doi: 10.3389/fendo.2023.1132915 (PMC10408669; doi:10.3389/fendo.2023.1132915)

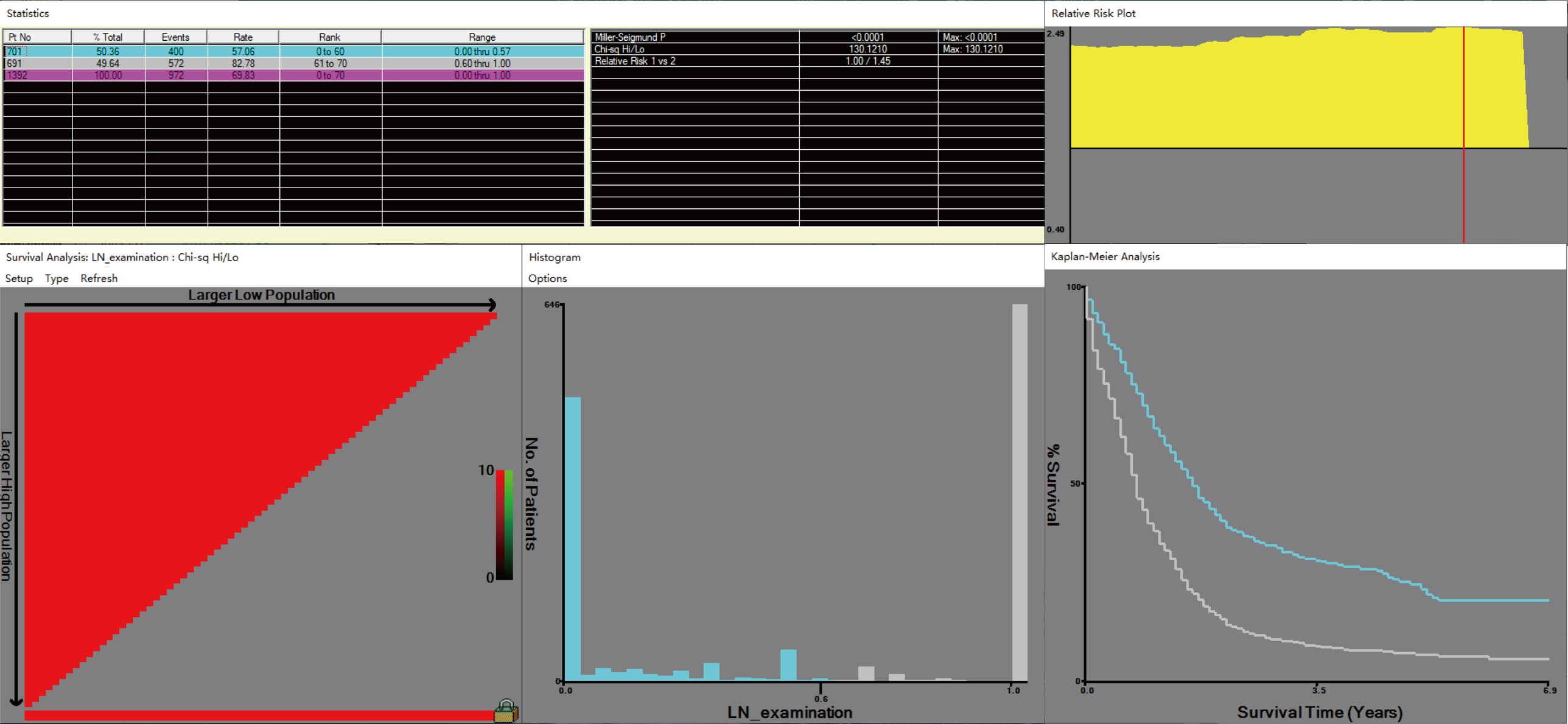

Supplement: Supplementary Figure 1 — The interface of X-tile categorizing LNR to achieve the optimal cut-off value. LNR, lymph node ratio. [file Image_1.jpeg]
